# Association of Aberrant DNA Methylation Level in the *CD4* and JAK-STAT-Pathway-Related Genes with Mastitis Indicator Traits in Chinese Holstein Dairy Cattle

**DOI:** 10.3390/ani12010065

**Published:** 2021-12-29

**Authors:** Tahir Usman, Nawab Ali, Yachun Wang, Ying Yu

**Affiliations:** 1 Key Laboratory of Agricultural Animal Genetics and Breeding, National Engineering Laboratory for Animal Breeding, College of Animal Science and Technology, China Agricultural University, Beijing 100193, China; wangyachun@cau.edu.cn; 2 College of Veterinary Science and Animal Husbandry, Abdul Wali Khan University, Mardan 23200, Pakistan; 3 Department of Zoology, Abdul Wali Khan University, Mardan 23200, Pakistan; nawabali79@gmail.com

**Keywords:** JAK-STAT pathway, DNA methylation, gene expression, epigenetic regulation, bovine mastitis resistance

## Abstract

**Simple Summary:**

Mastitis is the most common inflammatory disease of economic and animal welfare concern in dairy animals. The present study was designed to evaluate the gene expression and epigenetic modifications in cattle with mastitis and healthy cows. The CpG islands in the promoter regions of the *JAK2* and the *STAT5A* revealed hypo-methylation levels and higher gene expression in cows with mastitis compared to the healthy control, and vice versa in those with the *CD4 gene*. DNA methylation was negatively correlated with gene expression in the *JAK2* and *CD4* genes. Findings of the current study showed that aberrant DNA methylation due to mastitis in the promoter region of the three genes under study could be used as potential epigenetic markers to predict the mastitis susceptibility in dairy cattle.

**Abstract:**

The present study was designed to evaluate the gene expression and DNA methylation level in the promoter region of the *CD4* and the JAK-STAT-pathway-related genes. A total of 24 samples were deployed in the gene expression and 118 samples were used in the DNA methylation study. Student’s *t*-tests were used to analyze the gene expression and DNA methylation. The evaluation of DNA methylation in promoter regions of *JAK2* and *STAT5A* revealed hypo-methylation levels of CpG sites and higher gene expression in cows diagnosed with mastitis as compared to the healthy control, and vice versa in those with *CD4*. DNA methylation was negatively correlated with gene expression in *JAK2*, *STAT5A*, and *CD4* genes. Six, two, and four active transcription factors were identified on the CpG sites in the promoter regions of *JAK2*, *STAT5A*, and *CD4* genes, respectively. Regarding correlation analysis, the DNA methylation levels of *CD4* showed significantly higher positive correlations with somatic cell counts (*p* < 0.05). Findings of the current study inferred that aberrant DNA methylation in the CpG sites at the 1 kb promoter region in *JAK2*, *STAT5A*, and *CD4* genes due to mastitis in cows can be used as potential epigenetic markers to estimate bovine mastitis susceptibility in dairy cattle.

## 1. Introduction

Mastitis is an inflammation of the mammary gland and is characterized by pathological, physiological and bacteriological changes in the udder which affect the quality and quantity of the milk [1]. Mastitis is the most common inflammatory disease of economic and animal welfare concern in dairy animals. Globally, published estimates of the economic losses of clinical mastitis per cow on a farm range from EUR 61 to EUR 97 [2]. In addition to economic importance, bovine mastitis also carries public health significance. Milk and other dairy products are often reported to be contaminated with *S. aureus* and *E. coli*. Milk from cows with sub-clinical mastitis accidentally mixed into bulk milk enters food chain, thus causing a great threat to human health [3]. The California mastitis test and somatic cell count are tests routinely performed for the detection of mastitis in dairy animals [4]. Large numbers of pathogens causing mastitis; therefore, successful vaccination to control the disease is not effective. Generally, antimicrobial agents and management strategies are used to combat the disease in dairy cattle [5]. However, marker-assisted selection using genetics and epigenetics approaches is considered an appropriate strategy to minimize the incidence of mastitis in dairy cattle [6,7].

DNA methylation is a biochemical process in which a methyl group is added to the 5′-carbon of cytosine in the CpG dinucleotide sequence of DNA by the catalytic activity of the DNA methyltransferases, i.e., DNMT1, DNMT3A and DNMT3B [8]. The epigenetic markers of DNA are heritable during cell division, and do not alter the DNA sequence. Aberrant DNA methylation at CpG islands in the gene promoter region results in transcriptional silencing and is associated with many disease conditions, i.e., cancer formation and tumor progression [9]. Aberrant DNA methylation suppresses the transcription by inhibiting the binding of specific TFs [10]. Hypomethylation of global DNA methylation induced by *S. aureus* infection suppressed DNA methyltransferase activity in bovine mammary epithelial cells [11].

The JAK-STAT pathway mediates signal transduction between nucleus and the cell surface receptors [12], and any disturbance in this inflammatory signaling pathway can result in various immune disorders such as immune deficiency syndromes, various cancer conditions, and mastitis in dairy animals [13,14]. CpG sites in *JAK2* were hyper-methylated in myeloproliferative neoplasms compared with the healthy control, and 87.5% of the hyper-methylated CpG sites were located in the CpG island [15]. Our previous study demonstrated higher methylation levels of the *CD4* promoter region and lower gene expression in clinically mastitic cows compared with healthy controls using pyrosequencing assays and qRT-PCR [16]. CpG islands are CpG-rich areas in the promoter region of highly expressed genes and are a common site of methylation. Day and Bianco-Miotto (2013) [17] reported that CpG islands in around 55% of cases form clusters and result in the inhibition or activation of transcription depending on the status of methylation. DNA methylation is the most understood mechanism amongst all epigenetic mechanisms. DNA methylation markings have been suggested to be relatively stable over time in adult individuals, showing average heritability of 0.19 [18], and are reported to play key roles in the regulation of gene activity, DNA repair, recombination, replication, and the establishment and maintenance of cellular identity [18]. Studies on mouse models have shown that the risk of tumor incidence can be transmitted across generations (up to three generations) through aberrant DNA methylation [19,20]. Due to the stable nature of DNA methylation across generations and its average heritability of 0.18–0.19 [21], it has been suggested to consider it as a potentially important epigenetic marker for selection in breeding programs. Therefore, we designed this study to evaluate the role of DNA methylation levels at the 1 kb promoter region in *CD4* and JAK-STAT-pathway-related genes (*JAK2*, *STAT5A*, and *STAT5B*) in mastitic and healthy cows.

## 2. Materials and Methods

### 2.1. Sample Collection and DNA Extraction

Blood and milk samples were randomly collected from 118 lactating cattle (clinical mastitis, *n* = 58; healthy control, *n* = 60) from three Chinese Holstein dairy cattle farms (Qiqihar, Tianjin, and Shanxi) located in the northwest of China for DNA methylation analysis. The blood samples were collected in the morning between 9 a.m. and 11 a.m., whereas milk samples were collected at the routine milking time of 4 p.m. in the afternoon. A subset of 24 samples (of the total 118 samples) was used to evaluate the mRNA expression in the genes under study in mastitic and healthy cattle. We used routine dairy herd improvement (DHI) data to classify the different categories of lactating animal health status based on the SCC, i.e., healthy cows with SCC < 200,000/mL, subclinical mastitis cows with SCC ranging between 200,000 and 500,000/mL, and cows with clinical mastitis had SCC > 500,000/mL. The term “clinical mastitis” is used for cows with SCC > 500,000/mL based on the DHI data; however, these animals were apparently healthy and did not exhibit any clinical signs and symptoms. The healthy control represents lactating cows with healthy udders, without any history of mastitis in the last month, and SCC lower than 200,000/mL. The cows had different parities, ranging from parity one to five, and were milked three times per day (Table 1). The cattle were fed on a lactation diet as recommended by the Dairy Association of China for lactating cows (NRC2001). Milk samples were collected from all four quarters in sterile 50 mL falcon tubes and the SCC was analyzed at the official Dairy Center of China (Beijing, China). Blood samples were collected from the caudal vein of cows in three 9 mL tubes—one for serum isolation, the second for DNA extraction and the third for RNA extraction (the tubes contain pre-added TRIzol)—which were then immersed in liquid nitrogen at −196 °C to avoid any damage to the RNA. Serum was isolated from blood samples by incubating the samples at room temperature for 30 min followed by centrifugation at 3000× *g* for 10 min. The serum samples were then sent to Beijing Huaying Biological Technology Research Institute to evaluate serum biochemical assays of IL-4, IL-6, IL-10, IL-17, TNF-α, and IFN-γ using radioimmunoassay a technique, as described by Usman et al. (2017) [22].

### 2.2. DNA and RNA Extraction, cDNA Synthesis, and Real-Time Quantitative PCR

Genomic DNA and RNA extraction, reverse transcription of mRNA to cDNA, and real-time quantitative PCR were performed as described in our previous paper [14]. Primers were designed for *JAK2*, *STAT5A*, *CD4*, and *GAPDH* genes in Oligo 6.0 software, based on the golden rules for real-time PCR (Supplementary Materials Table S1) [23].

### 2.3. Bisulfite Treatment of Extracted DNA and Hot Start PCR

In order to evaluate the DNA methylation in *JAK2*, *STAT5A*, *STAT5B,* and *CD4* genes, we first checked the presence of CpG islands in the promoter region of these genes using a genome browser (UCSC BAU 6.0). We found a CpG island located in the 1 kb promoter region of all genes except for *STAT5B*. Sequencing primers of the *JAK2*, *STAT5A,* and *CD4* genes were designed with Oligo 6 software for evaluating DNA methylation in the genes under study (Supplementary Materials Table S2). Genomic DNA (1 μg) of each sample was treated for sodium bisulfite conversion using the EZ DNA Methylation Golden kit, following the manufacturer’s protocol (ZYMO Research, Irvine, CA, USA). A volume of 20 μL elution buffer was used to elute the bisulfite-converted DNA (ZYMO Research). In our study, we used a biotin-labeled universal primer (5′-GGGACACCGCTGATCGTTTA-3′), as mentioned in our previous research [23]. A hot-start PCR was performed to amplify the target sequence in a 25 μL volume, including 12.5 μL hot-start PCR premix (ZYMO Research), 0.5 μM forward primer, 0.05 μM reverse primer labeled with universal tail, 0.45 μM biotin-labeled universal primer, and 20 ng bisulfite-converted DNA. The PCR protocol was 95 °C for 10 min, 94 °C for 30 s, 50 to 60 °C for 45 s, and 72 °C for 45 s, and a final extension at 72 °C for 10 min for a total of 45 cycles. The PCR products were checked by running them on 2% agarose gel stained with ethidium bromide for visualization.

### 2.4. Quantitative DNA Methylation Evaluation Using Pyrosequencing

Pyrosequencing assays were conducted to quantitatively examine the methylation levels in the promoters of *JAK2*, *STAT5A*, and *CD4* genes in clinically mastitic cows and healthy controls. The DNA methylation level was tested for the nine CpG sites in the *JAK2* gene (USCS database: Bau6.0, Chr8: 39751199~39750253), seven CpG sites in the *STAT5A* gene (USCS database: Bau6.0, Chr19: 43033111~43034137), and five CpG sites located in the promoter region of the bovine *CD4* gene (USCS database: Bau6.0, Chr5: 104015622~104015994). Pyrosequencing techniques were used to evaluate the promoter DNA methylation levels using a Pyro Q-CpG system (Qiagen), following the manufacturer’s instructions. The high-quality purified DNA and the PCR products with high concentration (about 25 to 35 μL) were considered for pyrosequencing assays [24,25]. Streptavidin Sepharose High Performance (GE Healthycare) was utilized with the PCR products. The PCR products labeled with biotin and attached to Sepharose beads were distilled in 70% ethanol for 30 s, denatured for 30 s in denature buffer (0.2 M NaOH), then washed for 45 s with washing buffer with the Pyrosequencing Vacuum Prep Tool (Qiagen). Subsequently, 0.5 μM pyrosequencing primers of each gene was mixed with annealing buffer (Qiagen) in order to purify the PCR product. The methylation levels at each CpG site in the promoter region were revealed as the percentage [(^m^C/(^m^C + C)) × 100]. Here, ^m^C indicates methylated cytosine and C denotes unmethylated cytosine. In order to verify bisulfite conversion, non-CpG cytosine residues were used as internal controls.

### 2.5. Statistical Analysis

Student’s *t*-tests were used for analyzing the mRNA expression (values from RT-qPCR) and DNA methylation between mastitic cows and healthy controls. Pearson’s correlations of DNA methylation levels with mastitis-related traits in the genes under study were evaluated using the SAS (9.1) package. TFSEARCH software was used to predict the transcription factor binding sites in the promoter regions of the study genes. 

## 3. Results

### 3.1. CpG Sites Methylation in the Genes under Study

**Figure 1:**
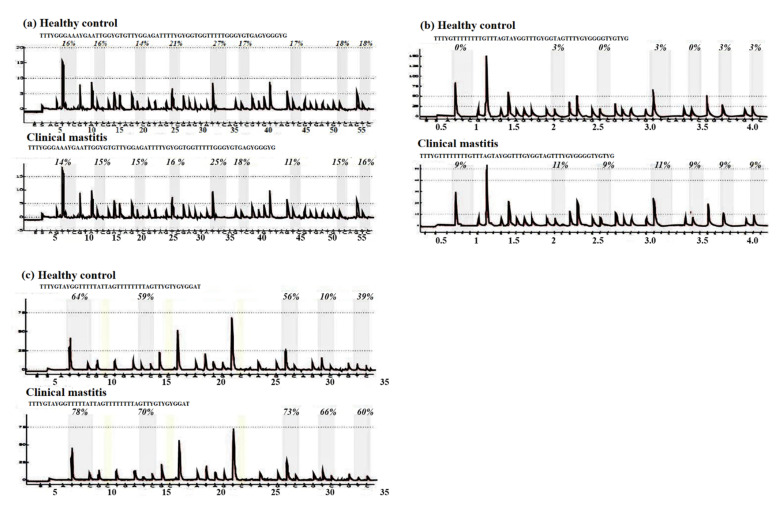
Results of bisulfite pyrosequencing showing aberrant methylation in clinically mastitic cow and healthy controls in almost all of the CpG sites in the genes under study: (**a**) *JAK2*, (**b**) *STAT5A*, (**c**) *CD4.*

The methylation statuses of CpG sites in the CpG island of the 1 kb promoter region in all genes under study were analyzed in mastitic cows and healthy controls using a pyrosequencing assay, except for the *STAT5B* gene, where no CpG island was present. The results of the pyrosequencing assay are shown below as methylation pyrogram (Figure 1). The pyrosequencing assay revealed aberrant methylation in almost all of the CpG sites in the genes under study (Table 2).

### 3.2. Predicted Binding Sites of TFs in the Promoter Regions of the Studied Genes 

**Figure 2:**
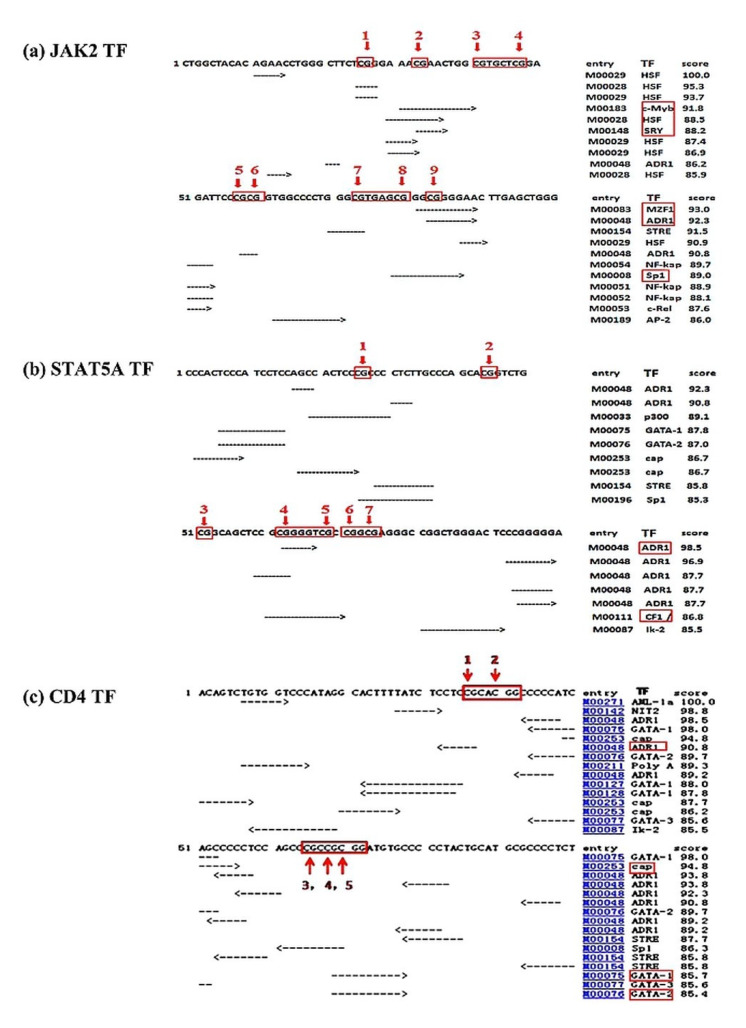
Predicted binding sites of transcription factors in the 1 kb promoter region of different genes under study: (**a**) *JAK2*, (**b**) *STAT5A*, (**c**) *CD4.* Number with red arrows indicate the CpG sites in the 1 kb promoter region of the studied genes. TF, predicted transcriptional factor.

The results showed six active transcription factors (TFs) (c-Myb, HSF, SRY, MZF1, ADR1, and Sp1) on the CpG sites of the *JAK2* gene, two TFs (ADR1 and Ik-2) on the *STAT5A* gene, and four active TFs (cap, Sp1, GATA-1, and GATA-2) were predicted on the promoter region of the *CD4* gene (Figure 2).

### 3.3. Relationship of DNA Methylation with mRNA Expression 

**Figure 3:**
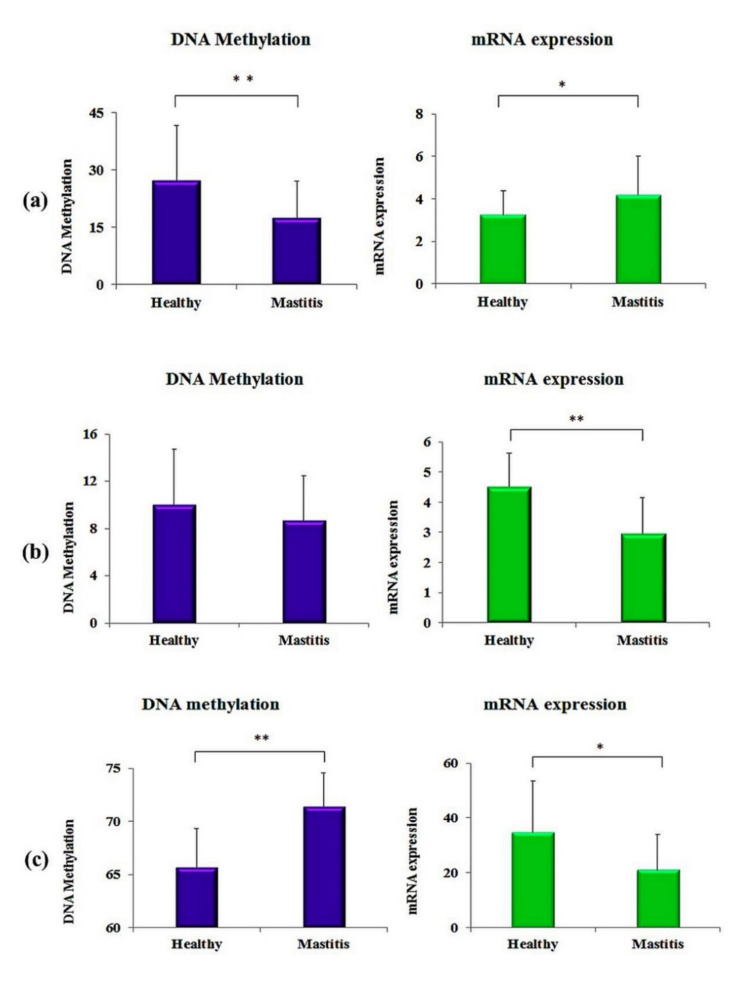
Results of CpG site methylation and mRNA expression in the clinically mastitic cows and in healthy controls: (**a**) *JAK2* gene, (**b**) *STAT5A* gene, (**c**) *CD4* gene. The asterisk “*” symbol shows the values are significant at (*p* < 0.05) and the “**” shows that values are significant at (*p* < 0.01).

To evaluate the results of DNA methylation with the transcription levels of the genes under study, we performed real-time quantitative PCR to estimate the mRNA expression levels. The results showed that the mRNA expression was significantly higher in clinically mastitic cows compare to the healthy controls in the *JAK2* gene, whereas in the *STAT5A* and *CD4* genes, the mRNA expression of mastitic cows was significantly lower than the healthy controls. Moreover, the mRNA expression and DNA methylation were negatively associated with *JAK2* and *CD4* genes, i.e., a higher methylation level was associated with a lower mRNA expression (Figure 3). DNA methylation in the figure below was calculated for the same cows for which mRNA expressions were analyzed.

### 3.4. Correlation among the CpG Sites in the Genes under Study

We analyzed the correlation among all of the CpG sites in CpG island of the genes under study. The results showed that almost all of the CpG sites were highly significantly correlated with each other in the *JAK2*, *STAT5A*, and *CD4* genes (Supplementary Materials Tables S3–S5).

### 3.5. Methylation Correlation with Mastitis Traits in the Studied Genes

The results of methylation correlations (Spearman) with mastitis indicator traits in *JAK2*, *STAT5A,* and *CD4* genes are shown in Table 3. The results indicated that the methylation levels of the *JAK2* gene exhibited a significant association with IL-4; *STAT5A* was significantly associated with IL-4 and Il-17; and the methylation level of the *CD4* gene was highly significantly associated with SCC, SCS, mastitis status, and IFN-γ (*p* < 0.01).

## 4. Discussion

Unlike genetic variants, which cause irreparable changes in the gene and can potentially result in gene activation or other effects, epigenetic modifications are known to be reversible changes which influence gene expression whilst keeping the DNA sequence unaltered [8]. DNA methylation is the most understood mechanism amongst all of the epigenetic mechanisms and its role is well known in mediating the gene expression [18]. DNA methylation is known to be an important epigenetic modification that potentially suppresses gene expression; thus, it can play a vital role in inflammatory conditions [17,18,19]. In recent years, the locus-specific methylation levels in peripheral leukocytes have emerged as suitable epigenetic markers in breast cancer [26] and in various other inflammatory conditions [17,18,19]. It has been reported that DNA methylation patterns can serve as a stable epigenetic marker of gene silencing memory in animals [19]. One recent study suggested potential regulatory roles of DNA methylation in bovine mammary glands during *S. aureus*-induced mastitis [27]. Song et al. (2016) reported aberrant DNA methylation in 1078 genes in cows with subclinical mastitis vs. healthy controls; most of these genes were associated with inflammation and ErbB signaling pathway [28]. A study in mice showed that DNA methylation is quite stable across generations and the epigenetic information can be passed on for up to three generations [19,20]. Due to the stable nature of DNA methylation across generations and its average heritability of 0.18–0.19 [21], it is suggested to consider it as an important epigenetic marker for selection in breeding programs. Based on the important function of DNA methylation in various inflammatory conditions in different species, its role as a potential epigenetic marker in the 1 kb promoter region of genes under study in the mastitis resistance of dairy cattle has been evaluated.

We found that the methylation levels at CpG sites in the *JAK2* gene in mastitic cows was significantly low and the mRNA expression was significantly higher, and vice versa in healthy controls (*p* < 0.05). This revealed that a lower methylation status of the *JAK2* gene in mastitic cows possibly induces enhanced gene expression. Perez et al. (2013) reported that CpG sites in *JAK2* were aberrantly methylated in myeloproliferaitve neoplasms compared with healthy controls, and 87.5% of the differentially methylated CpG sites were located in the CpG island [15]. *JAK2* plays an essential role in the activation of the lmo2 leukemogenic gene through phosphorylation of the H3Y41 histone [29]. The results of the present study are in line with a previous study which reported that the methylation patterns of CpG sites in the *JAK2* gene were hypomethylated after 24 h in *E. coli*-challenged porcine mammary epithelial cells compared with the control cells [30]. It is well documented that hyper-methylation of the CpG island is associated with decreased mRNA expression because hyper-methylated CpG sites can block the transcription factor (TF) motifs [31,32]. The presence of six active TFs in the CpG island of the *JAK2* gene in the present study shows that these TFs could have a potential role in gene activation and silencing; thus, they can play important roles in mastitis resistance. The TFs revealed in a recent study were reported to be involved in transcription activation and gene regulation [33]. The variable DNA methylation levels of CpG sites in the *JAK2* promoter region reveal that methylation at these sites could be a potential epigenetic marker for mastitis resistance. We suggest that further studies should be carried on epigenetic modifications (DNA methylation) of the *JAK2* gene in dairy cattle with clinical mastitis in a larger population. In the *STAT5A* promoter region, the CpG sites exhibited higher methylation in healthy controls than mastitic cows, and the association between mRNA expression and methylation was non-significant. Moreover, the presence of the two active transcription factors in the CpG sites of the *STAT5A* gene manifest that these TFs can potentially play an important role in gene switch-on or switch-off. Stefanowicz et al. (2012) reported variable methylation between airway epithelial cells and the peripheral blood mononuclear cells [34]. DNA methylation selectively inhibits gene expression in the *STAT5A* gene of oncogenic cells [35]. To the best of our knowledge, this is the first epigenetic study focused on DNA methylation level evaluations in mastitic cows and healthy controls in the *STAT5A* gene. The results of the different DNA methylation levels, although non-significant, between the mastitic cows and the healthy controls, suggest that *STAT5A* could be considered in future epigenetic studies on mastitis resistance in dairy cattle.

The findings of the present study are in complete agreement with our previous study that reported hyper-methylation and lower mRNA expression in the promoter region of the *CD4* gene in mastitic cows compared with healthy controls [16]. CpG island methylation in the *CD4* promoter region was downregulated in a line of chickens susceptible to MDV, whereas the gene expression was upregulated in the spleen of that particular line of chicken [36]. Many studies have reported that DNA methylation influences *CD4* gene silencing and plays an important role in inflammatory conditions, causing the development and differentiation of *CD4*^+^ T cells [37]. Li et al. (2010) reported that the hyper-methylation of CpG sites can suppress gene expression by inactivating the transcription factor binding sites [31]. The presence of four active TFs at CpG sites of the *CD4* gene indicates that these TFs might be involved in the switching on/off of the *CD4* gene [31]. Hence, these results are promising, suggesting that DNA methylation at the promoter region of *CD4* can be considered as a potential epigenetic marker for mastitis resistance. We recommend carrying out further research to verify the functional role of DNA methylation in promoter CpG islands of the *CD4* gene in mastitis resistance studies.

The results of the present study are in line with our previous study [16], although the time, individuals’ samples, and the environmental conditions were totally different between the two studies. These results indicate that *CD4* DNA methylation at the promoter region can be used as a potential biomarker in mastitis resistance studies. Similarly, the study of *JAK2* and *STAT5A* methylation status, and mRNA expression in mastitic versus healthy controls, indicated the potential role of the CpG island at the 1 kb promoter region in these genes in hypo-methylation, and higher mRNA expression in mastitic cows compared to healthy controls in the *JAK2* gene. The results from TFSEARCH software revealed active transcription factors in the CpG sites of these genes, which indicates that variation in the TF methylation during mastitis can affect the switch-on or switch-off of the gene, thus modifying gene action.

## 5. Conclusions

The aberrant DNA methylation levels in *JAK2* and *CD4* genes between mastitic cows and healthy controls and their inverse relationship with gene expression suggests that these genes could be potential candidate genes of epigenetic importance. The CpG sites at the CpG island in the 1 kb promoter region of the studied genes showing peculiar methylation patterns are recommended to be studied in other populations at larger scale to validate their roles as potential biomarkers in future epigenetic studies on mastitis resistance in dairy cattle.

## Figures and Tables

**Table 1:** Information of the parity and health status of the samples used in the study.

	Clinical Mastitis	Healthy Control
Number	58	60
SCC *	>500,000	<200,000
Parity range	1–5	1–5

* SCC, somatic cell count.

**Table 2:** DNA methylation levels of CpG sites in 1 kb promoter regions of the *JAK2*, *STAT5A*, and *CD4* genes in clinically mastitic and healthy cows.

CpG Position	Gene	Clinical Mastitis (Mean ± SE)	Healthy Control (Mean ± SE)	*p* Value
CpG site 1	*JAK2*	15.6 ± 10.1	25.9 ± 15.7	0.05
	*STAT5A*	10.3 ± 5.7	12.9 ± 12.5	0.40
	*CD4*	82.7 ± 7.8	75.8 ± 5.3	4 × 10^−3^
CpG site 2	*JAK2*	14.7 ± 6.4	25.5 ± 11.5	3 × 10^−4^
	*STAT5A*	8.9 ± 10.2	10.1 ± 6.9	0.02
	*CD4*	68.6 ± 3.0	66 ± 2.5	7 × 10^−3^
CpG site 3	*JAK2*	13.9 ± 5.6	20.9 ± 9.9	3 × 10^−3^
	*STAT5A*	8.8 ± 1.7	12.6 ± 8.2	0.15
	*CD4*	77.8 ± 4	69.5 ± 5	2 × 10^−5^
CpG site 4	*JAK2*	19 ± 12	36.5 ± 22	4 × 10^−3^
	*STAT5A*	10.3 ± 5.6	10.7 ± 11.9	0.50
	*CD4*	70.1 ± 6.3	62.1 ± 3.9	1 × 10^−4^
CpG site 5	*JAK2*	25.9 ± 17.7	46.6 ± 28.5	6 × 10^−3^
	*STAT5A*	5.5 ± 1.2	7.2 ± 1.2	0.02
	*CD4*	57.8 ± 3.3	54.9 ± 5.7	0.05
CpG site 6	*JAK2*	14.8 ± 6.7	23.9 ± 10.5	2 × 10^−3^
	*STAT5A*	7.4 ± 1.5	9 ± 2.2	0.01
CpG site 7	*JAK2*	15.2 ± 8	21.8 ± 8.5	0.01
	*STAT5A*	8.5 ± 5.9	15.3 ± 5.5	0.11
CpG site 8	*JAK2*	17.2 ± 9	24.3 ± 9.6	7 × 10^−^^3^
CpG site 9	*JAK2*	19.6 ± 8.5	24.1 ± 11.7	0.07
All (Mean)	*JAK2*	17.5 ± 9.6	27.3 ± 14.3	3 × 10^−3^
	*STAT5A*	8.7 ± 3.8	10 ± 5.7	0.20
	*CD4*	71.4 ± 3.2	65.7 ± 3.7	5 × 10^−5^

**Table 3:** Spearman’s correlation of DNA methylation levels with mastitis-related traits in *JAK2*, *STAT5A*, and *CD4* genes.

Traits	*JAK2* Methylation	*STAT5A* Methylation	*CD4* Methylation
SCC	−0.06	0.17	0.67 **
*p* value	0.55	0.33	5 × 10^−4^
SCS	−0.05	0.15	0.71 **
*p* value	0.61	0.41	3 × 10^−5^
Status	−0.13	−0.24	0.66 **
*p* value	0.18	0.16	2 × 10^−5^
IL-4	0.28 *	−0.48 *	−0.15
*p* value	0.03	0.03	0.51
IL-6	−0.04	−0.51*	−0.10
*p* value	0.73	0.03	0.65
IL-10	−0.08	0.14	−0.24
*p* value	0.56	0.56	0.31
IL-17	−0.19	0.44 *	0.15
*p* value	0.15	0.05	0.53
TNF-α	0.07	0.08	−0.22
*p* value	0.63	0.73	0.36
IFN-γ	0.11	−0.01	−0.54 **
*p* value	0.41	0.95	0.01

The asterisk “*” symbol shows the values are significant at (*p* < 0.05), and the “**” shows that values are significant at (*p* < 0.01). SCC, somatic cell count; SCS, somatic cell score; Status, health status; IL-4, interleukin 4; IL-6, interleukin-6; IL-10, interleukin-10; IL-17, interleukin-17; TNF-α, tumor necrosis factor-alpha; IFN-γ, interferon-γ.

## Data Availability

All the data is included in the manuscript files and the Supplementary Files of this research paper.

## Supplementary Materials

The following additional supporting information may be found online at https://www.mdpi.com/article/10.3390/ani12010065/s1 for this article: Table S1. Information of primers used for real-time PCR; Table S2. Information of primers used for pyrosequencing assay; Table S3. Correlation amongst nine CpG sites in the JAK2 gene; Table S4. Correlation amongst seven CpG sites in the STAT5A gene; Table S5. Correlation amongst five CpG sites in the CD4 gene.
