# Supplementary material for: Association of Aberrant DNA Methylation Level in the CD4 and JAK-STAT-Pathway-Related Genes with Mastitis Indicator Traits in Chinese Holstein Dairy Cattle
Source: Animals (Basel). 2021 Dec 29;12(1):65. doi: 10.3390/ani12010065 (PMC8749870; doi:10.3390/ani12010065)
Supplement: Supplementary file 1 [file animals-12-00065-s001.zip › animals-1485414-supplementary.pdf]

**Table S1.** Information of primers used for real-time PCR.

| Gene | mRNA Primer Sequencing | Product length (bp) |
|---|---|---|
| ***JAK2*** | Forward: TGAAGACCGAGACCCTACAC | 212 |
| | Reverse: CTGCAAGGATTTAAGGATTTC | |
| ***STAT5A*** | Forward: GGTAGTTCTTCTGCTGGGAT | 106 |
| | Reverse: CATTCTCTGTGTCCTGCGTG | |
| ***CD4*** | Forward: AGCAGAAAGTGAAACTCGTGG | 128 |
| | Reverse: ACCAACTTCGGCTGATTTGAG | |
| ***GADPH*** | Forward: AGATGGTGAAGGTCGGAGTG | 200 |
| | Reverse: CGTTCTCTGCCTTGACTGTG | |

**Table S2.** Information of primers used for pyrosequencing assay.

| Gene | DNA Methylation Sequencing | Product length (bp) |
|---|---|---|
| ***JAK2*** | Forward: GTTTTGTTTATTGTAGAGAT | 295 |
| | Reverse: GGGACACCGCTGATCGTTTAAAACTAACTCACTCCCAACC | |
| | Sequencing: GATTGGTTATATAGAATTTGG | |
| | Assay: TTTYGGGAAAYGAATTGGYGTGTTYGGAGATTTTYGYGGT GGTTTTTGGGYGTGAGYGGGYGG | |
| ***STAT5A*** | Forward: TTGATAGATGAGAAAATTGAG | 325 |
| | Reverse: GGGACACCGCTGATCGTTTACTACCTCTAACAAACCATACAC | |
| | Sequencing: TTTATTTTTATTTTTTAGTTAT | |
| | Assay: TTTYGTTTTTTTTGTTTAGTAYGGTTTGYGGTAGTTTYGYGGGGT YGTYG | |
| ***CD4*** | Forward: GTTGGGGGGAAGAGGGTATAG | 193 |
| | Reverse: GGGACACCGCTGATCGTTTAAAATCCCTTCAAAAAACCATATCTC | |
| | Sequencing: TTTATAGGTATTTTTATTTT | |
| | Assay: TTTYGTAYGGTTTTTATTAGTTTTTTTTAGTTYGTYGYGGAT | |

**Table S3.** Correlation amongst nine CpG sites in the *JAK2* gene.

| | CPG1 | CPG2 | CPG3 | CPG4 | CPG5 | CPG6 | CPG7 | CPG8 | CPG9* |
|---|---|---|---|---|---|---|---|---|---|
| CPG1 | . | 0.93 (0.0001) | 0.89 (0.0001) | 0.95 (0.0001) | 0.95 (0.0001) | 0.88 (0.0001) | 0.77 (0.01) | 0.83 (0.0001) | 0.79 (0.0001) |
| CPG2 | 0.93 (0.0001) | . | 0.93 (0.0001) | 0.91 (0.0001) | 0.92 (0.0001) | 0.89 (0.0001) | 0.87 (0.0001) | 0.92 (0.0001) | 0.79 (0.0001) |
| CPG3 | 0.89 (0.0001) | 0.93 (0.0001) | . | 0.89 (0.0001) | 0.89 (0.0001) | 0.87 (0.0001) | 0.90 (0.0001) | 0.91 (0.0001) | 0.72 (0.0001) |
| CPG4 | 0.95 (0.0001) | 0.91 (0.0001) | 0.89 (0.0001) | . | 0.94 (0.0001) | 0.98 (0.0001) | 0.71 (0.01) | 0.73 (0.0001) | 0.77 (0.0001) |
| CPG5 | 0.95 (0.0001) | 0.92 (0.0001) | 0.89 (0.0001) | 0.94 (0.0001) | . | 0.92 (0.0001) | 0.76 (0.01) | 0.81 (0.0001) | 0.85(0.0001) |
| CPG6 | 0.88 (0.0001) | 0.89 (0.0001) | 0.87 (0.0001) | 0.98 (0.0001) | 0.92 (0.0001) | . | 0.76 (0.01) | 0.79 (0.0001) | 0.89 (0.0004) |
| CPG7 | 0.77 (0.01) | 0.87 (0.0001) | 0.90 (0.0001) | 0.71 (0.01) | 0.76 (0.01) | 0.76 (0.01) | . | 0.91 (0.0001) | 0.37 (0.16) |
| CPG8 | 0.83 (0.0001) | 0.92 (0.0001) | 0.91 (0.0001) | 0.73 (0.0001) | 0.81 (0.0001) | 0.76 (0.01) | 0.91 (0.0001) | . | 0.55 (0.05) |
| CPG9 | 0.79 (0.0001) | 0.79 (0.0001) | 0.72 (0.0001) | 0.77 (0.0001) | 0.85(0.0001) | 0.89 (0.0004) | 0.37 (0.16) | 0.55 (0.05) | . |

*The values in brackets ( ) show the *P* value.

**Table S4.** Correlation amongst seven CpG sites in the *STAT5A* gene.

| | CpG1 | CpG2 | CpG3 | CpG4 | CpG5 | CpG6 | CpG7* |
|---|---|---|---|---|---|---|---|
| | | 0.93 | 0.92 | 0.95 | 0.85 | 0.83 | 0.81 |
| CpG1 | . | (0.001) | (0.001) | (0.001) | (0.01) | (0.01) | (0.01) |
| | 0.93 | | 0.93 | 0.91 | 0.82 | 0.79 | 0.77 |
| CpG2 | (0.001) | . | (0.001) | (0.001) | (0.01) | (0.01) | (0.01) |
| | 0.92 | 0.93 | | 0.91 | 0.81 | 0.79 | 0.72 |
| CpG3 | (0.001) | (0.001) | . | (0.001) | (0.01) | (0.01) | (0.05) |
| | 0.95 | 0.91 | 0.91 | | 0.64 | 0.71 | 0.77 |
| CpG4 | (0.001) | (0.001) | (0.001) | . | (0.15) | (0.05) | (0.05) |
| | 0.85 | 0.82 | 0.81 | 0.64 | | 0.82 | 0.69 |
| CpG5 | (0.01) | (0.01) | (0.01) | (0.15) | . | (0.01) | (0.05) |
| | 0.83 | 0.79 | 0.79 | 0.71 | 0.82 | | 0.76 |
| CpG6 | (0.01) | (0.01) | (0.01) | (0.05) | (0.01) | . | (0.01) |
| | 0.81 | 0.77 | 0.72 | 0.77 | 0.69 | 0.76 | |
| CpG7 | (0.01) | (0.01) | (0.05) | (0.05) | (0.05) | (0.01) | . |

*The values in brackets ( ) show the *P* value.

**Table S5.** Correlation amongst five CpG sites in the *CD4* gene.

| | CpG1 | CpG2 | CpG3 | CpG4 | CpG5* |
|---|---|---|---|---|---|
| | | 0.48 | 0.71 | 0.69 | 0.17 |
| CpG 1 | . | (0.006) | (0.0001) | (0.0001) | (0.34) |
| | 0.48 | | 0.51 | 0.36 | 0.57 |
| CpG2 | (0.006) | . | (0.004) | (0.05) | (0.0009) |
| | 0.71 | 0.51 | | 0.86 | 0.44 |
| CpG3 | (0.0001) | (0.004) | . | (0.0001) | (0.01) |
| | 0.69 | 0.36 | 0.86 | | 0.38 |
| CpG4 | (0.0001) | (0.05) | (0.0001) | . | (0.03) |
| | 0.17 | 0.57 | 0.44 | 0.38 | |
| CpG5 | (0.34) | (0.0009) | (0.01) | (0.03) | . |

*The values in brackets ( ) show the *P* value.
